# Where do HIV-infected adolescents go after transfer? – Tracking transition/transfer of HIV-infected adolescents using linkage of cohort data to a health information system platform

**DOI:** 10.7448/IAS.20.4.21668

**Published:** 2017-05-16

**Authors:** Mary-Ann Davies, Priscilla Tsondai, Nicki Tiffin, Brian Eley, Helena Rabie, Jonathan Euvrard, Catherine Orrell, Hans Prozesky, Robin Wood, Dolphina Cogill, Andreas D. Haas, Annette H. Sohn, Andrew Boulle

**Affiliations:** ^a^ Centre for Infectious Disease Epidemiology and Research (CIDER), School of Public Health and Family Medicine, University of Cape Town, Cape Town, South Africa; ^b^ Western Cape Province Department of Health, Health Impact Assessment Directorate, Cape Town, South Africa; ^c^ Red Cross War Memorial Children’s Hospital and Department of Paediatrics and Child Health, University of Cape Town, Cape Town, South Africa; ^d^ Department of Paediatrics and Child Health, Tygerberg Academic Hospital, University of Stellenbosch, Stellenbosch, South Africa; ^e^ Khayelitsha ART Programme and Médecins Sans Frontières, Khayelitsha, Cape Town, South Africa; ^f^ Gugulethu HIV Programme and Desmond Tutu HIV Centre, University of Cape Town, Cape Town, South Africa; ^g^ Division of Infectious Diseases, Department of Medicine, University of Stellenbosch and Tygerberg Academic Hospital, Cape Town, South Africa; ^h^ Institute of Social and Preventive Medicine (ISPM), University of Bern, Bern, Switzerland; ^i^ TREAT Asia/amfAR – The Foundation for AIDS Research, Bangkok, Thailand

**Keywords:** HIV-1, adolescents, transfer, transition, sub-Saharan Africa, antiretroviral, data linkage

## Abstract

**Introduction**: To evaluate long-term outcomes in HIV-infected adolescents, it is important to identify ways of tracking outcomes after transfer to a different health facility. The Department of Health (DoH) in the Western Cape Province (WCP) of South Africa uses a single unique identifier for all patients across the health service platform. We examined adolescent outcomes after transfer by linking data from four International epidemiology Databases to Evaluate AIDS Southern Africa (IeDEA-SA) cohorts in the WCP with DoH data.

**Methods**: We included adolescents on antiretroviral therapy who transferred out of their original cohort from 10 to 19 years of age between 2004 and 2014. The DoH conducted the linkage separately for each cohort and linked anonymized data were then combined. The primary outcome was successful transfer defined as having a patient record at a facility other than the original facility after the transfer date. Secondary outcomes included the proportion of patients retained, with HIV-RNA <400 copies/ml and CD4 > 500 cells/µl at 1, 2 and 3 years post-transfer.

**Results**: Of 460 adolescents transferred out (53% female), 72% transferred at 10–14 years old, and 79% transferred out of tertiary facilities. Overall, 81% of patients transferred successfully at a median (interquartile range) of 56 (27–134) days following transfer date; 95% reached the transfer site <18 months after transfer out. Among those transferring successfully, the proportion retained decreased from 1 to 3 years post-transfer (90–84%). There was no significant difference between transfer and 1–3 years post-transfer in the proportion of retained adolescents with HIV-RNA <400 copies/ml and CD4 > 500 cells/µl except for HIV-RNA <400 copies/ml at 3 years (86% vs. 75%; *p* = 0.007). The proportion virologically suppressed and with CD4 > 500 cells/µl was significantly lower at 1 and 2 years post-transfer in those transferring at 15–19 vs. 10–14 years of age. Using laboratory data alone over-estimated time to successful transfer.

**Conclusions**: Linking cohort data to health information system data allowed efficient assessment of post-transfer outcomes. Although >80% of adolescents transferred successfully with nearly 85% of them retained for 3 years post-transfer, the decline in the proportion virologically suppressed and poorer outcomes in older adolescents are concerns.​

## Introduction

There were an estimated 1.8 million HIV-infected adolescents globally in 2015 and this population is increasing both due to longer survival of perinatally HIV-infected children as well as behaviourally transmitted infections at older ages [1]. With the decline in new infant infections due to widespread coverage of effective prevention of mother-to-child transmission, the burden of paediatric HIV is shifting into adolescence [2,3]. Some studies have reported poor retention and virologic response in adolescents initiating HIV care and antiretroviral therapy (ART) in resource-limited settings (RLS) [4–7]. It is therefore important to evaluate long-term outcomes in HIV-infected adolescents. However, collection of such data may be challenging as adolescents frequently transfer care to a different facility [8]. For example, in the International epidemiology to Evaluate AIDS-Southern Africa (IeDEA-SA) cohort collaboration, one in four adolescents transferred care between 10 and 13 years of age [9]. In wealthy countries, adolescent HIV care transfer usually involves moving from specialist paediatric HIV care to an adult clinic and occurs when the adolescent reaches approximately 18–25 years of age, coinciding with the developmental shift towards adulthood, hence being referred to as “adolescent transition” [10]. Developmentally, adolescents take more responsibility for their own decision-making and self-care, but it is also a time when risk-taking behaviour tends to increase. Cohorts from Europe and North America report concerning outcomes regarding retention, mortality and virologic suppression when HIV-infected adolescents transition to adult care, especially among those behaviourally infected or moving to a clinic at a different facility [11–13]. There are very limited data on HIV-infected adolescents transitioning to adulthood in RLS [8]. In Thailand, 73% of 67 adolescents were retained in care between 1 and 6 years following transition to adult care at the same institution with nearly 80% having HIV-RNA<40 copies/ml [14]. While dedicated adolescent services in RLS report high retention and virologic suppression [15], a transition clinic in Uganda found only one-third of patients used services regularly, with especially poor utilization in those not on ART [16].

In contrast to the model of care in resource-rich countries, in RLS, healthcare for HIV-infected children is decentralized and mostly occurs in non-specialist primary care facilities, with clinical officers, medical officers and nurses being responsible for paediatric care [17–19]. Where children do initiate HIV care and ART at a specialist paediatric facility, they are frequently transferred from the paediatric facility to primary care during childhood or early adolescence once stable on ART [20]. This is not unique to HIV care – in RLS including in the Western Cape Province (WCP) of South Africa, children with a range of conditions are usually transferred out of paediatric care when they reach approximately 13 years of age. Hence, while patients are leaving the specialist paediatric care environment, this may not coincide with the time at which they transition to adulthood and take responsibility for their own self-care.

**Figure 1.**
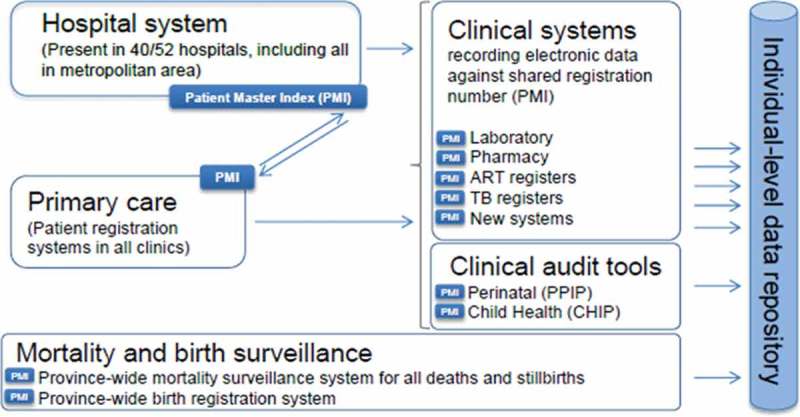
Sharing a unique health identifier (PMI) to enable subsequent linkage of patient data across multiple domains.

In RLS, children receiving ART at primary care facilities have been shown to have comparable or better retention and virologic suppression compared to those at specialist facilities [19,21,22]. Among young children transferring from tertiary to primary care in the WCP, more than 80% successfully reached the transfer site with improved CD4 and viral load responses after transfer [23]. However, there is little data on transition/transfer outcomes of adolescents in RLS where transfer may occur earlier during adolescence than in wealthy countries. This lack of data is partly because of the challenge of following adolescents after transfer has occurred. The WCP Department of Health (DoH) is unique in that many of its patient information systems are uniform throughout the jurisdiction. These systems capture data on visits to most health facilities in the province, clinical data on laboratory, pharmacy, ART and tuberculosis, as well as mortality and birth surveillance (Figure 1). In order to track outcomes across disease programmes and facilities, the WCP DoH has developed a Provincial Health Data Centre (PHDC) and patients use the same health identifier (folder number) in all services. IeDEA-SA includes four cohorts providing paediatric HIV care in the WCP. The WCP PHDC therefore provides a unique opportunity to examine patient outcomes after transfer by linking IeDEA-SA cohort data collected at each site to data from the PHDC information systems. Data linkage provides an efficient and valuable means to track outcomes in these patients, especially as on-the-ground tracing studies are resource intensive and challenging, so infrequently conducted in RLS. We aimed to (i) assess the feasibility of this linkage approach for examining transfer outcomes in HIV-infected adolescents transferring care between 10 and 19 years of age and (ii) describe the following outcomes: successful transfer to a different facility as well as retention within the WCP health service, viral suppression and CD4 response for up to 3 years after transfer.

## Methods

### Study population

We included data from the four IeDEA-SA cohorts providing paediatric HIV care in WCP – two tertiary care (Tygerberg Academic Hospital and Red Cross War Memorial Children’s Hospital) and two primary care (Gugulethu and Khayelitsha Community Health Centres) cohorts. These are all urban Cape Town cohorts in both formal and informal settlement areas. We included all adolescents on ART if they had a valid WCP DoH folder number and were recorded as transferred out by the above cohorts between 10 and <20 years of age from March 2004 through December 2014. IeDEA-SA cohorts collect routine patient monitoring data on demographics, clinical outcomes and laboratory tests. These data are de-identified and transferred annually to the IeDEA-SA Data Centres at the Universities of Cape Town, South Africa, and Bern, Switzerland, for inclusion in combined analyses using a standard data transfer format. All cohorts have ethics approval to examine long-term outcomes of patients at their facilities through linkage to other datasets and to contribute de-identified data to the IeDEA Data Centre. Waivers of informed consent have been granted by the respective institutional review boards as the analyses use only anonymized data that are already collected as part of routine patient care. The IeDEA Data Centres have ethics approval to combine and conduct analyses on the de-identified data.

### Patterns of paediatric HIV care transfer in the Western Cape

There are various patterns of patient transfer in the context of paediatric HIV care outlined below. Patients transferring for any of the reasons below would be documented as “transferred out” in the IeDEA-SA data.

- Most common is transfer of stable patients from tertiary care specialist paediatric facilities to primary care facilities where paediatric care is largely through clinical nurse practitioners or medical officers [20,24]. While such transfer may coincide with later adolescence and transition to adulthood, it most often occurs in childhood or early adolescence. Nevertheless, these children and young adolescents leave the protected and dedicated paediatric care environment of a specialist facility to the frequently busier and more generalist environment of primary care where they are seen alongside adults, and adolescent patients may need to assume more responsibility for self-care. This pattern of transfer is very frequent in the context of the decentralized model of HIV care in WCP where patients should receive care at the facility closest to their homes and the lowest level appropriate to their disease condition [20].
- Patients may transfer from one primary care facility to another within WCP due to patient migration or choice, or due to ART programmes starting in facilities closer to the patient’s home.
- Patients who are unwell or experiencing treatment failure may be transferred from primary care up to tertiary care to receive specialist management.
- Patients may transfer to a different province due to patient migration.

### Western Cape PHDC and linkage process

Most patient information systems are uniform throughout the WCP public sector health service platform with the same health identifier (folder number) being used for each patient in all services. The shared folder number and related patient details are referred to as the Patient Master Index (PMI), which is hosted by a common information system used at all major provincial hospitals and linked to all primary care and HIV services, laboratory, and pharmacy systems through a web-services interface (Figure 1). All laboratory test results are digitized, and all hospitals have electronic dispensing. The PHDC, which operates within a Microsoft SQL Server environment, receives daily updates from these different electronic data sources, and integrates the datasets using the PMI to link the different records to individuals. This provides an up-to-date clinical record per individual for facility encounters, laboratory tests undertaken and drugs dispensed, regardless of the facility of origin.

To conduct the linkage, each cohort securely submitted to the PHDC a list of folder numbers of adolescents who had transferred out of their facility. The data were linked to PHDC data (facility visits; admissions; CD4 and viral load tests; pharmacy records of antiretroviral drugs dispensed). All PHDC data linkages using identified data were performed by PHDC staff who are bound by South African provincial and national requirements around protection of patient confidentiality. The PHDC performs de-duplication analyses to identify situations where one individual has been assigned more than one PMI in error. Where such linkage can be conclusively demonstrated, outcomes data were separately provided for linked PMI numbers, allowing for inclusion of complete de-duplicated clinical data for these individuals. Once linkage was complete, data were securely returned to the individual cohorts with patient identifying information stored separately from clinical data. Each cohort linked the clinical data to the demographic data using study-specific identifiers, then de-identified the combined data and submitted the anonymized dataset to the IeDEA Data Centre for analysis.

### Outcomes and analysis

The primary outcome was the proportion of patients with “successful transfer” defined as a record (visit, laboratory test or pharmacy) at a facility other than the original facility after the transfer-out date at the original facility. We also examined the proportion of patients with successful transfer within 18 months of the transfer-out date from the original facility. Secondary outcomes included the following:

- Transfer delay defined as the gap in days between transfer-out  date and first record indicating successful transfer.
- Proportion of patients retained, proportion with HIV-RNA/CD4 measured, virologically suppressed (HIV-RNA <400 copies/ml) and with CD4 absolute count >500 cells/µl at 1, 2 and 3 years post-transfer according to age at transfer (10–14 vs. 15–19-years old).

We examined outcomes after transfer by requesting each contributing site to link routinely collected data for transferred patients to PHDC information systems prior to transferring the anonymized data to the IeDEA-SA Data Centre. The PHDC included data through mid-October 2016. We considered patients “retained” if they had ≥1 visit within 6 months on either side of the time point evaluated i.e. for retention at 1 year, an adolescent had to have ≥1 visit between (transfer in date +365 days) 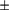
6 months. When assessing retention for each year following the transfer in date in those who successfully transferred, we only included adolescents with sufficient potential follow-up after successful transfer and before the date of PHDC database closure for the outcome to be evaluated. For example, when assessing retention 2 years after transfer out, we only included adolescents who had successfully transferred at least 2.5 years before PHDC database closure. Therefore, the number of patients in whom retention and laboratory outcomes can be assessed is lower at 2 and 3 years after transfer. HIV-RNA and CD4 measures at each duration following transfer were the measures taken on the date closest to the date of successful transfer plus 1, 2 or 3 years, respectively, within a window of 6 months on either side of the time point. We compared the proportion of patients considered to have transferred successfully and the transfer delay when using different sources of data (laboratory, pharmacy and visits) to assess the value of different data sources when examining transfer outcomes.

All analyses were conducted in Stata13 (College Station, Texas). We described characteristics of children at transfer using medians with interquartile ranges and proportions for continuous and categorical variables, respectively. As route of infection was not recorded, we considered children perinatally infected if they enrolled in HIV care before 13 years of age. We compared laboratory values at transfer with laboratory values at each time point after transfer using the Wilcoxon sign rank test for median CD4 count and binomial tests for the proportion with HIV-RNA <400 copies/ml, respectively. Each comparison was limited to children with values measured both at transfer and the respective time point. We examined predictors of successful transfer using adjusted logistic regression. We included the following variables in the model a priori: likely perinatal infection (indicated by enrolment in HIV care at <13 years of age), sex, age at transfer (10–14 years vs. 15–19 years), HIV-RNA <400 copies/ml at transfer and whether the patient was transferring out of primary or tertiary care.

## Results

### Patient characteristics at transfer

A total of 460 adolescents 10–19 years (53% female) were transferred out of their original site during the study period (Table 1). All patients had recorded folder numbers and could be linked to PHDC data, except in the smallest cohort, where only 33 adolescents transferred and 9 of them (27%) had valid recorded folder numbers and could be included in the study. Patients transferring from that site with folder numbers were similar to patients without folder numbers except that they were more likely to be female (*p* = 0.073). Most transfers were from tertiary care facilities (79%) with 72% of transfers during early adolescence (<15 years of age). Ninety per cent of adolescents were considered perinatally infected using the definition of enrolment in HIV care before 13 years of age. If we restricted the definition of “perinatally infected” to adolescents who enrolled in HIV care before 10 years of age, 69% would be considered “perinatally infected”. However, most children enrolling between 10 and 12-years old did so at tertiary care paediatric clinics and had advanced disease suggesting longstanding/perinatal infection. At transfer 78% of adolescents were virologically suppressed and 64% had CD4 >500 cells/µl.

**Table 1.** Characteristics of adolescents transferring between 10 and 19 years of age

	Transferred successfully	Not transferred successfully	All adolescents
Number (%)	374 (81.3)	86 (18.7)	460 (100)
Sex			
Male (*n*, %)	174 (46.5)	41 (47.7)	215 (46.7)
Female (*n*, %)	200 (53.5)	45 (52.3)	245 (53.3)
Age at enrolment*			
<13 years (*n*, %)	338 (90.4)	72 (83.7)	410 (89.1)
≥13 years (*n*, %)	36 (9.6)	14 (16.3)	50 (10.9)
Age at transfer			
<15 years (*n*, %)	267 (71.4)	65 (75.6)	332 (72.2)
15–19 years (*n*, %)	107 (28.6)	21 (24.4)	128 (27.8)
Cohort at transfer			
Primary care	59 (15.8)	36 (41.9)	95 (20.7)
Tertiary care	315 (84.2)	50 (58.1)	365 (79.3)
HIV-RNA <400 copies/ml at transfer (*n*/*N*, %)	289/355 (81.4)	49/79 (62.0)	338/434 (77.9)
CD4 ≥ 500 cells/µl at transfer (*n*/*N*, %)	243/367 (66.2)	44/79 (55.7)	287/446 (64.3)
Median (IQR) age at ART start (years)	8.1 (5.3–10.7)	9.3 (6.6–11.7)	8.4 (5.4–10.9)
Median CD4 count at ART start (cells/µl)	299 (169–554)	241 (144–467)	289 (161–537)
Median CD4 % at ART start	13.0 (7.1–20.0)	12.7 (7.7–19.4)	12.8 (7.1–19.8)
Median (IQR) age at transfer (years)	12.9 (11.4–15.3)	12.5 (11.4–15.0)	12.8 (11.4–15.3)
Median (IQR) years on ART at transfer	6.1 (1.7–8.3)	3.9 (1.1–6.9)	5.5 (1.5–8.2)
Median CD4 count at transfer (cells/µl)	643 (411–918)	524 (227–739)	636 (387–876)

*proxy for perinatally infected

### Successful transfer and outcomes after transfer

**Figure 2.**
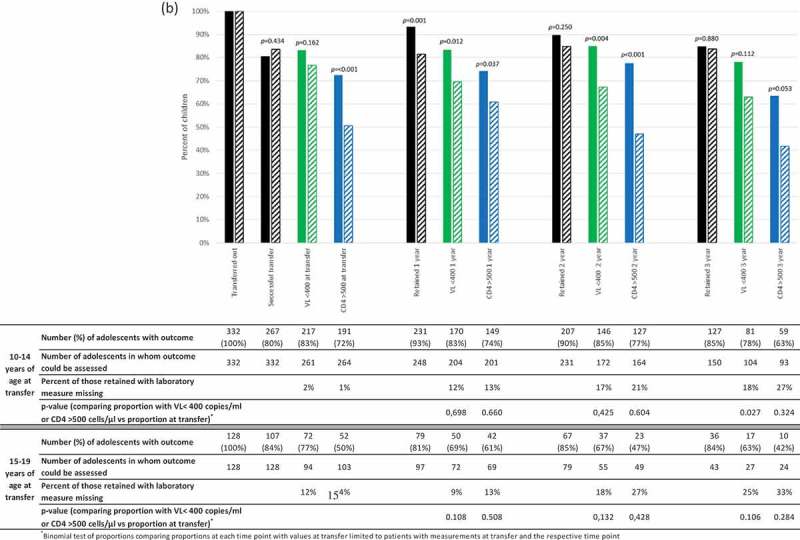
a) Per cent of all children successfully transferred and retained at 12, 24 and 36 months after successful transfer and percent with HIV-RNA <400 copies/ml and CD4 > 500 cells/µl among those retained. b) Per cent of all children successfully transferred and retained at 12, 24 and 36 months after successful transfer according to age group at transfer (10-14 years [solid bars] and 15-19 years [diagonal striped bars]). Percent with HIV-RNA <400 copies/ml and CD4 > 500 cells/µl among those retained. P-values comparing outcomes in each age group using chi2 tests are shown on the graph.

Using linked visit, laboratory and pharmacy data, 81% (95%CI: 77–85%) of children were considered to transfer successfully, of whom 95% linked to the transfer site within 18 months of their transfer date (Figure 2). As the intended transfer site was not recorded in the cohort database, we could not exclude patients who had intended to transfer out of the province or to the private sector from our analysis. These estimates therefore likely represent lower bound estimates of transfer success, since we only linked to patient data in WCP public sector. From previous analyses of transfers in children, about 6% of transfers are outside of the province [23] so we could assume that at least 85% (82% of approximately 95% of patients transferring within the province) successfully transfer. Median (interquartile range) follow-up after successful transfer was 3.3 (2.2–4.9) years.

**Figure:**
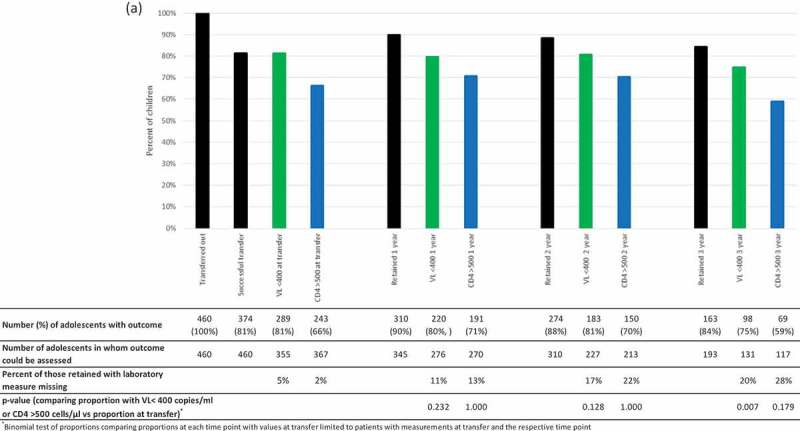

Following transfer, there is a small drop-off in retention overall from 90% (95% CI: 86–93%) at 1 year to 84% (95% CI: 79–89%) at 3 years (Figure 2a). Retention was lower in 15–19-year olds vs. 10–14-year olds at 1 and 2 years but similar at 3 years (Figure 2b). Of note, in 15–19-year olds retention was higher at 2 and 3 years after transfer compared to 1 year after transfer. This could represent a gap in care following successful transfer with later re-engagement, or could be ascribed to improving information systems such as electronic dispensing being expanded over the last several years. The proportion with HIV-RNA <400 copies/ml and CD4 >500 cells/µl of those assessed was similar in years 1 and 2 but decreased at 3 years after transfer from 80% (95%CI: 75–84%) to 75% (95%CI: 67–82%) and 71% (95%CI: 65–76%) to 59% (95%CI: 50%-68%), respectively (Figure 2a). However, these proportions were not significantly lower than the proportion at transfer when comparing proportions in patients with values available at both time points except for HIV-RNA <400 copies/ml at 3 years (86% vs. 75%; *p* = 0.007) (Figure 2a).

The proportion with HIV-RNA <400 copies/ml and CD4 >500 cells/µl at 1, 2 and 3 years post transfer was consistently lower in adolescents who were older at transfer. Although not statistically significant at 3 years this is likely due to lack of power because of the small number of older adolescents with sufficient potential follow-up and laboratory measures available for analysis (Figure 2b). The median (IQR) CD4 count declined significantly from 654 (444–926) cells/µl at transfer to 639 (461–903) at 2 years (*p* = 0.034) and 580 (429–793) at 3 years (*p* = 0.004) (analysis restricted to patients with CD4 measured both at transfer and the respective time post-transfer). The proportion of patients with missing laboratory test measurements increased between 1 and 3 years post successful transfer (from 11–20% [HIV-RNA] and 13–28% [CD4 count]) (Figure 2a). Nevertheless, among those retained in care but with missing HIV-RNA at 3 years, 38% had a subsequent measurement shortly after the 3-year window with HIV-RNA <400 copies/ml and a further 19% had measurements at 2 years and had been continually suppressed. Only 13% had subsequent HIV-RNA values that were not suppressed, while the remaining 31% with no subsequent HIV-RNA measurements either had no previous measurements or had not been previously continuously suppressed. The increasing proportion with missing CD4 values is in keeping with South African treatment guidelines which, since 2013, recommended that CD4 monitoring should not be done in clinically stable and virologically suppressed patients [25]. In the 22 patients who had CD4 missing but did have HIV-RNA measured, 21 were either virologically suppressed or had been previously continuously suppressed with this being the first non-suppressed HIV-RNA, so CD4 measurement would not have been indicated.

### Predictors of successful transfer

Adolescents transferring out of tertiary care facilities, those ≥15 years of age and virologically suppressed at transfer were more likely to transfer successfully (Table 2). In contrast, there was no significant difference in transfer success by likely route of infection.

**Table 2.** Logistic regression of predictors of successful transfer (adjusted for all other variables in the table)​

Characteristic	Adjusted Odds Ratio	95% CI	*p*-value
Gender			
Male	1		
Female	1.20	0.70–2.04	0.506
Original cohort			
Tertiary care	1		
Primary care	0.28	0.16–0.50	<0.001
VL at transfer			
≥400 copies/ml	1		
<400 copies/ml	2.75	1.58–4.80	<0.001
Age at enrolment*			
≥13 years	1		
<13 years	2.85	1.14–7.15	0.026
Age at transfer			
10–14 years	1		
15–19 years	1.06	1.01–1.11	0.017

*proxy for perinatal infection

**Table 3.** Comparison of proportion of children considered successfully transferring and the transfer delay using data from different data sources

	Proportion transferring successfully	Proportion transferring successfully within 18 months	Median (IQR) days between last contact at original site and first contact at transfer site
Visits only	80%	72%	73 (28–197)
Laboratory results only	73%	63%	241 (142–388)
Pharmacy records only*	54%	29%	451 (56–1161)
Laboratory, pharmacy and visits	81%	77%	56 (27–134)

*Low proportion with pharmacy records due to non-availability of electronic dispensing at all facilities throughout the study period.

### Comparison of estimated successful transfer using different data sources

The proportion of children who would be estimated to transfer successfully using different PHDC data sources is shown in Table 3. Using laboratory records alone, 73% of children would be considered to transfer successfully with 63% linking to the referral site within 18 months of the transfer out date. Corresponding values using visit records alone were 80% and 72%, respectively, and 81% and 77% using all data sources. The median (IQR) transfer delay was lower using visit data than laboratory data (73[28–197] vs. 241[142–388] days), and 56 (27–134) days using all data sources.

## Discussion

This is one of the first studies examining adolescent outcomes after transfer in RLS. In terms of transfer and post-transfer outcomes, overall, >80% of adolescents aged 10–19 years transferred successfully and retention in those who transferred remained relatively high for up to 3 years (85%). Nevertheless, the proportion of adolescents with HIV-RNA <400 copies/µl and median CD4 count declined by 3 years following successful transfer, and outcomes were consistently worse in older adolescents. We found that assessment of transfer success and long term outcomes in adolescents is feasible using HIV cohort data linked to health information system data such as the PHDC. Almost all transferring adolescents had valid folder numbers, data were prepared by the cohorts and linkage conducted by the PHDC in <4 weeks, and a very high proportion of patients could be linked to PHDC data.

### Comparison with other studies

Patient outcomes following transfer in our study compare favourably with studies of transfer and adolescent transition from wealthy countries, although transfer in our study occurred at younger ages and cannot be considered equivalent to transition to adult care. In this respect, the poorer retention, viral load and CD4 outcomes following transfer in those transferring between 15 and 19 years of age in our study is a key concern. A recent US study of patients transferring between 21 and 25 years of age reported only 50% of patients retained at one year post-transfer [12]. In the UK, retention of perinatally HIV-infected adolescents transitioning to adult care at 17 years of age was higher in those transferring to adult clinics at the same facility (92%) compared to those moving to a new facility (72%) [13]. An Italian study reported 84% retention following transfer in children and adolescents 0–18-years old [26].

There are very few studies of transition/transfer outcomes in adolescents in RLS. A study in Thailand that transitioned adolescents to adult care in small groups rather than individually reported 73% retention between 1 and 6 years post-transfer [14]. In our study, assuming about 5% of adolescents transfer out of WCP [23], success within the province is at least 85%, with nearly 85% retention of the transferred group 3 years later, giving overall retention of 72% – very similar to the Thailand study. Virologic suppression was also similar at approximately 80% in both our study and the Thailand one [14].

It is understandable that virologically suppressed adolescents are more likely to successfully link to transfer sites as virologic suppression is a measure of adherence. However, it is unclear why children transferring out of primary care were less likely to transfer successfully. This may be because a higher proportion of adolescents transferring out of primary care are transferring out of the province, or because a major reason to transfer out of primary care is because an adolescent is unwell or has treatment failure warranting specialist care, both of which are associated with mortality and non-retention.

### Strengths and limitations

The major strength of this study was linkage of well-curated cohort data with PHDC data providing a very efficient way to assess transfer and long-term outcomes in adolescents that is not facility centric. In the absence of this linkage, follow-up of these adolescents would be censored at the last visit at the original site, with substantial challenges to tracking long term outcomes. This method has broad applicability; it could also be used to assess whether patients lost to follow-up have silently transferred to a different facility and to examine other health outcomes such as pregnancy incidence in HIV-infected adolescents. In particular, the use of a combination of different data sources (laboratory, visit and pharmacy) enhanced outcome ascertainment and laboratory records alone would have over-estimated the transfer delay substantially. To our knowledge, this is the first analysis of transfer outcomes during adolescence in sub-Saharan Africa. Given increasing decentralization and task-shifting in paediatric ART programmes across the sub-continent [19] and the growing numbers of HIV-infected adolescents, transfer during adolescence will likely become more frequent so understanding these outcomes and identifying efficient ways to track adolescents after transfer is important.

A major limitation of our study is that linkage was limited to WCP as unique identifiers are not used nationally, so we could not assess transfers outside the province. Similarly, the intended transfer site was not routinely recorded by the original cohort so we do not have an accurate measure of the proportion of transfers out of WCP, and could not assess whether patients transferred to the intended site within the province. While use of data from only administrative sources such as the PHDC allowed us to efficiently track post-transfer outcomes, our analysis had to be limited to variables collected by routine health information systems. Hence, we could not assess the impact of other key variables such as socio-economic status, mental health and adherence on post-transfer outcomes. In addition, we did not have an additional non-administrative data source (such as a tracing study), so could not assess whether gaps in care identified in our study were real or artefactual due to incomplete coverage of a particular data source. Overall, only a small number of patients transferred especially during late adolescence and the proportion of likely behaviourally infected adolescents was small, limiting our ability to comprehensively assess transfer outcomes in these groups. There were relatively small numbers of adolescents with sufficient long term potential follow-up (i.e. transferred long enough before PHDC database closure) to assess outcomes at 3 years after transfer. We did not have national identity numbers of the adolescents so did not link to the mortality registry and were unable to determine whether mortality is an important reason for non-retention. Nevertheless, at least 30–45% of patients not retained in a particular year were not deceased as they returned to care in the subsequent year, and we have previously shown that mortality ascertainment in children <15 years of age (the majority of our study) is much higher than in adults [27]. In keeping with the aims of the review to examine outcomes following formal transfer to another facility, and the inclusion criteria, we only examined outcomes in patients that were formally documented as transferred out in the IeDEA-SA data. Since transfer of paediatric and adolescent patients is a key component of the model of HIV care in the Western Cape, documentation of transfer is generally reliable. However, it is possible that some patients who transferred were not correctly coded and thus excluded from our study. In addition, we did not examine “silent transfer” where patients appear LTFU at the original facility but have themselves resumed care elsewhere without being formally transferred.

Finally, the completeness of HIV-RNA testing decreased over time limiting assessment of HIV-RNA suppression. Reasons for reduced completeness of HIV-RNA testing are unclear; it is possible that HIV-RNA testing is not prioritized in patients who have been stable and virologically suppressed on ART for a long time or that visit spacing is greater in stable patients with fewer opportunities to test HIV-RNA. The fact that nearly 60% of patients with missing HIV-RNA data at 3 years post-transfer either had subsequent values indicating suppression or were previously continuously suppressed would support this. It is possible that adolescents who were unwell or known to have poor adherence underwent laboratory testing more frequently, biasing results towards poorer outcomes. Nevertheless, the finding that completeness of routine HIV-RNA testing decreased at longer follow-up durations is important in itself.

## Conclusions

Our study demonstrates the enormous potential for assessing long-term outcomes of adolescents using linked health information system data such as the PHDC. The proportion of adolescents with successful transfer and retention for up to 3 years was reasonable overall and comparable with other studies from RLS. Nevertheless, the outcomes of those not retained need to be explored. The decline in virologic suppression and poorer outcomes in older adolescents are concerns.

## References

[1] UNAIDS AIDSinfo Online Database [cited 2016 7 1]. Available from http://www.aidsinfoonline.org/devinfo/libraries/aspx/Home.aspx ​

[2] Johnson L, Davies M, Moultrie H, Sherman G, Bland R, Rehle T, et al The effect of early initiation of antiretroviral treatment in infants on pediatric AIDS mortality in South Africa - A model-based analysis. Pediatr Infect Dis J. 2012;31:474–24. 22189531 10.1097/INF.0b013e3182456ba2

[3] Maskew M, Bor J, MacLeod W, Carmona S, Sherman G, Fox MP. The youth treatment bulge in South Africa: increasing numbers, inferior outcomes among adolescents on ART. *International AIDS Conference* Durban South Africa; 2016.

[4] Evans D, Menezes C, Mahomed K, Macdonald P, Untiedt S, Levin L, et al Treatment outcomes of HIV-infected adolescents attending public-sector HIV clinics across Gauteng and Mpumalanga, South Africa. AIDS Res Hum Retroviruses. 2013;29:892–900. 23373540 10.1089/aid.2012.0215 PMC3653371

[5] Lamb MR, Fayorsey R, Nuwagaba-Biribonwoha H, Viola V, Mutabazi V, Alwar T, et al High attrition before and after ART initiation among youth (15-24 years of age) enrolled in HIV care. Aids. 2014;28:559–68. 24076661 10.1097/QAD.0000000000000054 PMC4517438

[6] Weigel R, Estill J, Egger M, Harries AD, Makombe S, Tweya H, et al Mortality and loss to follow-up in the first year of ART: Malawi National ART Programme. Aids. 2012;26:365–73. 22095194 10.1097/QAD.0b013e32834ed814 PMC3811026

[7] Nachega JB, Hislop M, Nguyen H, Dowdy DW, Chaisson RE, Regensberg L, et al Antiretroviral therapy adherence, virologic and immunologic outcomes in adolescents compared with adults in southern Africa. J Acquir Immune Defic Syndr. 2009;51:65–71. 19282780 10.1097/QAI.0b013e318199072e PMC2674125

[8] Sohn AH, Hazra R The changing epidemiology of the global paediatric HIV epidemic: keeping track of perinatally HIV-infected adolescents. J Int AIDS Soc. 2013;16:18555. 23782474 10.7448/IAS.16.1.18555 PMC3687075

[9] Davies M, Sawry S, Phiri S, Rabie H, Eley B, Fatti G, et al What does transfer mean in SSA? Patterns of transfer in Southern African perinatally HIV-infected adolescents. *International AIDS Conference* Durban, South Africa; 2016.

[10] Hussen SA, Chahroudi A, Boylan A, Camacho-Gonzalez AF, Hackett S, Chakraborty R Transition of youth living with HIV from pediatric to adult-oriented healthcare: a review of the literature. Future Virol. 2015;9:921–9. 25983853 10.2217/fvl.14.73 PMC4433446

[11] Fish R, Judd A, Jungmann E, O’Leary C, Foster C Mortality in perinatally HIV-infected young people in England following transition to adult care: an HIV Young Persons Network (HYPNet) audit. HIV Med. 2014;15:239–44. 24112550 10.1111/hiv.12091

[12] Ryscavage P, Macharia T, Patel D, Palmeiro R, Tepper V Linkage to and retention in care following healthcare transition from pediatric to adult HIV care. AIDS Care. 2016;28:561–5. 26766017 10.1080/09540121.2015.1131967

[13] Hope RL, Judd A, Foster C, Prime K, Tookey P, Jungmann E, et al Clinical outcomes in adults with perinatal HIV after transfer from pediatric care. Abstract presented at: *Conference on Retroviruses and Opportunistic Infections* Boston, USA; 2016.

[14] Hansudewechakul R, Pongprapass S, Kongphonoi A, Denjanta S, Watanaporn S, Sohn AH Transition of Thai HIV-infected adolescents to adult HIV care. J Int AIDS Soc. 2015;18:20651. 26637523 10.7448/IAS.18.1.20651 PMC4670453

[15] Zanoni B, Cairns C, Sibaya T, Haberer J High retention and viral suppression rates in a dedicated adolescent-friendly HIV clinic in South Africa. *8th International Workshop on HIV Pediatrics* Durban, South Africa; 2016.

[16] Nyabigambo A, Muliira JK, Atuyambe L, Babikako HM, Kambugu A, Ndoleriire C Determinants of utilization of a no-cost HIV transition clinic: a cross-sectional study of young adults living with HIV/AIDS. Adolesc Health Med Ther. 2014;5:89–99. 24966709 10.2147/AHMT.S57950 PMC4043429

[17] Bolton-Moore C, Mubiana-Mbewe M, Cantrell RA, Chintu N, Stringer EM, Chi BH, et al Clinical outcomes and CD4 cell response in children receiving antiretroviral therapy at primary health care facilities in Zambia. Jama. 2007;298:1888–99. 17954540 10.1001/jama.298.16.1888

[18] Sutcliffe CG, Van Dijk JH, Bolton C, Persaud D, Moss WJ Effectiveness of antiretroviral therapy among HIV-infected children in sub-Saharan Africa. Lancet Infect Dis. 2008;8:477–89. 18652994 10.1016/S1473-3099(08)70180-4

[19] Penazzato M, Davies MA, Apollo T, Negussie E, Ford N Task shifting for the delivery of pediatric antiretroviral treatment: a systematic review. J Acquir Immune Defic Syndr. 2014;65:414–22. 24583614 10.1097/QAI.0000000000000024

[20] Eley B, Nuttall J Antiretroviral therapy for children: challenges and opportunities. Ann Trop Paediatr. 2007;27:1–10. 17469726 10.1179/146532807X170448

[21] Fatti G, Bock P, Eley B, Mothibi E, Grimwood A Temporal trends in baseline characteristics and treatment outcomes of children starting antiretroviral treatment: an analysis in four provinces in South Africa, 2004-2009. J Acquir Immune Defic Syndr. 2011;58:e60-7. 21857355 10.1097/QAI.0b013e3182303c7e

[22] Morsheimer M, Dramowski A, Rabie H, Cotton M Paediatric ART outcomes in a decentralised model of care in Cape Town, South Africa. South Afr J HIV Med. 2014;15:148–53.

[23] Arowosegbe O, Davies M, Apolles P, Boulle A, Eley B Outcomes of children transferring out of a specialist pediatric clinic using linkage to laboratory data. *International Workshop on HIV Pediatrics* Durban, South Africa; 2016.

[24] Davies M, Keiser O, Technau K, Eley B, Rabie H, Van Cutsem G, et al Outcomes of the South African National Antiretroviral Treatment (ART) programme for children - The IeDEA Southern Africa Collaboration. S Afr Med J. 2009;99:730–7. 20128272 PMC2925142

[25] South African National Department of Health Revised antiretroviral treatment guideline 2013. Houghton, MI; 2013 ​

[26] Righetti A, Prinapori R, Nulvesu L, Fornoni L, Viscoli C, Di Biagio A Transitioning HIV-infected children and adolescents into adult care: an Italian real-life experience. J Assoc Nurses AIDS Care. 2015;26:652–9. 26116060 10.1016/j.jana.2015.05.003

[27] Johnson LF, Dorrington RE, Laubscher R, Hoffmann CJ, Wood R, Fox MP, et al A comparison of death recording by health centres and civil registration in South Africans receiving antiretroviral treatment. J Int AIDS Soc. 2015;18:20628. 26685125 10.7448/IAS.18.1.20628 PMC4684576

